# Biparatopic HER2-targeted nanobody binder synergizes with trastuzumab in resistant tumor cells

**DOI:** 10.3389/fimmu.2025.1711448

**Published:** 2025-10-27

**Authors:** Xinlin Liu, Li Guo, Yihuan Wang, Xiangzheng Meng, Yunhan Shao, Xinyi Fan, Cong Wang, Wenjing Zhu, Jingyu Cao, Peng Sun

**Affiliations:** ^1^ Department of Hepatobiliary and Pancreatic Surgery, The Affiliated Hospital of Qingdao University, Qingdao, China; ^2^ Qingdao Cancer Institute, Qingdao, China; ^3^ Qingdao Municipal Hospital, University of Health and Rehabilitation Sciences, Qingdao, China; ^4^ School of Basic Medicine, Qingdao Medical College, Qingdao University, Qingdao, China; ^5^ Department of Allergy, The Affiliated Hospital of Qingdao University, Qingdao, China; ^6^ Medical Research Department, Qingdao Hospital, University of Health and Rehabilitation Sciences (Qingdao Municipal Hospital), Qingdao, China

**Keywords:** HER2, biparatopic antibody, nanobody, resistance, synergistic efficacy

## Abstract

Human epidermal growth factor receptor 2 (HER2) is a key oncogenic driver in diverse solid tumors. Although HER2-targeted therapies such as trastuzumab and pertuzumab confer substantial clinical benefits, therapeutic resistance remains a major challenge, necessitating the development of next-generation agents. Here, we engineered a biparatopic nanobody-based binder, A9F5-H2F5-Fc (AH), designed to target ECD I and ECD II of HER2. In HER2-expressing tumor cells, AH induced greater receptor saturation, internalization, and degradation than the combination of trastuzumab and pertuzumab. Notably, in trastuzumab-resistant cancer cells, AH exhibited superior synergistic antitumor efficacy in combination with trastuzumab, outperforming trastuzumab plus pertuzumab. Structural modeling predicted a *trans*-binding mode that enables multivalent HER2 clustering, indicative of a distinct mechanism of action. These findings highlight AH as a rationally designed biparatopic binder with potential to overcome trastuzumab resistance and underscore the potential of nanobody-based biparatopic strategies to enhance antitumor efficacy in HER2-positive cancers.

## 1 Introduction

Human epidermal growth factor receptor 2 (HER2), a member of the epidermal growth factor receptor (EGFR/HER) family, is a transmembrane tyrosine kinase receptor that is overexpressed in approximately 20–30% of breast and gastric cancers, as well as in subsets of other solid tumors (1). HER2 amplification drives oncogenic signaling pathways that promote tumor progression, foster therapeutic resistance, and correlate with poor clinical outcomes (2–4). In contrast to other EGFR family members, HER2 functions as an orphan receptor, lacking a known ligand, and is activated through heterodimerization with its counterparts (5, 6). Over the past three decades, HER2-targeted therapies have revolutionized the treatment landscape of HER2-positive solid tumors (2, 7). A range of HER2-directed agents, including small-molecule tyrosine kinase inhibitors (TKIs), monoclonal antibodies (mAbs), and antibody-drug conjugates (ADCs), have been developed and approved for clinical use.

Trastuzumab, the first approved mAb targeting extracellular domain (ECD) IV of HER2, exerts antitumor activity by inhibiting ligand-independent HER2-HER3 dimerization, inducing cell cycle arrest, and mediating antibody-dependent cell-mediated cytotoxicity (ADCC) (8). Despite improving clinical outcomes in HER2-positive breast cancers, many patients ultimately develop resistance and experience disease recurrence (9, 10). Pertuzumab, a mAb targeting the dimerization arm (DA) of HER2-ECD II, was developed to block ligand-dependent HER2 heterodimerization (11). Leveraging their complementary mechanisms of action (MOAs), the combination of pertuzumab, trastuzumab, and chemotherapy has demonstrated superior antitumor efficacy compared to trastuzumab plus chemotherapy in metastatic, adjuvant, and neoadjuvant settings, leading to the FDA approval of pertuzumab as a synergistic partner (12, 13). However, the clinical success of this dual-antibody strategy in breast cancer has not translated to other HER2-positive tumors (3). In HER2-positive metastatic gastric or gastro-esophageal junction cancer, the addition of pertuzumab failed to significantly improve overall survival over trastuzumab plus chemotherapy (14). Furthermore, intrinsic and acquired resistance to existing HER2-targeted therapies remains a major clinical obstacle, underscoring the need for next-generation anti-HER2 agents.

Mechanisms of resistance include receptor downregulation, HER2 heterodimerization with other EGFR family members, compensatory activation of downstream signaling pathways, and HER2 alterations (15, 16). Bispecific antibodies (bsAbs), which are designed to target two epitopes or antigens, possess multiple MOAs not achievable by conventional monospecific antibodies, rendering them promising candidates for overcoming resistance (17). For instance, zenocutuzumab, a bsAb against HER2 and HER3, potently inhibits heregulin (HRG)-driven signaling of HER2-HER3 heterodimerization and has received accelerated FDA approval for patients with *NRG1* fusion-positive non-small cell lung cancer (NSCLC) or pancreatic adenocarcinoma (18). As a subclass of bpAbs, biparatopic antibodies (bpAbs) bind non-overlapping epitopes on the same target and confer unique MOAs (19). Several anti-HER2 bpAbs, such as zanidatamab and anbenitamab, have shown encouraging efficacy in preclinical and early clinical studies (20–24). Current HER2-targeted bpAbs in clinical development are derived from trastuzumab and pertuzumab, and thus rely on epitopes within ECD II and ECD IV (25). However, oncogenic mutations in HER2-ECD—particularly S310F in ECD II—can impair pertuzumab binding and compromise therapeutic efficacy (26). Consequently, the clinical utility of bpAbs dependent on the pertuzumab epitopes may be limited in patients harboring such mutations. Recent studies suggest that targeting ECD I or ECD III may synergize with existing anti-HER2 therapies (27–31). We previously developed a nanobody-Fc fusion protein targeting ECD I that exhibited significantly enhanced trastuzumab-synergistic antitumor efficacy in trastuzumab-resistant models (32). These findings highlight the potential of targeting alternative HER2 epitopes to overcome the resistance.

Nanobodies, or single variable heavy-chain domains (VHHs), are the smallest naturally occurring antigen-binding fragments, characterized by high solubility, stability, and low immunogenicity (33–37). Unlike IgG antibodies, nanobodies can recognize antigens in the absence of a light chain (38). Their monomeric structure makes them ideal scaffolds for bsAb construction, as they avoid chain mispairing and undesired self-association (39, 40). Here, we report the generation of a biparatopic nanobody-based binder, A9F5-H2F5-Fc (AH), composed of two HER2-specific nanobodies fused in tandem to an IgG Fc region. This tetravalent binder targets ECD I and ECD II of HER2, achieves high binding saturation, and promotes HER2 internalization and degradation. Moreover, AH exhibited significantly enhanced synergy with trastuzumab in resistant tumor models.

## 2 Methods

### 2.1 Cell lines, antibodies, and biological material

The human cell lines (NCI-N87, MCF-7, JIMT-1, SKBR3, and BT474) were obtained from ATCC. Expi293 cells were purchased from Thermo Fisher Scientific. Trastuzumab and pertuzumab were produced in-house using established protocols. A non-specific IgG antibody (Beyotime, A7001) was used as a negative control.

### 2.2 Recombinant protein expression and purification

To construct biparatopic binders, sequences encoding anti-HER2 nanobodies were cloned into the pSCSTa expression vector with either a C-terminal or N-terminal Fc tag. The nanobody-based binders were expressed in Expi293 cells. The pSCSTa vector was transiently transfected into Expi293 cells. After 7 days of culture, the supernatant was collected and then purified by Protein A affinity chromatography using AT Protein A Diamond Plus (BestChrom, AA402305). The eluted proteins were dissolved in PBS buffer. Protein concentrations were determined using the BCA assay, and purity was assessed by SDS-PAGE.

Chimeric HER2-ECD proteins (HER2-mD1, HER2-mD2, HER2-mD3, and HER2-mD4) were constructed as previously described (32). Briefly, the ECD I (T23-R217), ECD II (T218-C342), ECD III (Y344-A510), and ECD IV (C511-T652) of the HER2 protein were substituted with the corresponding regions from the murine homolog. The DNA sequences encoding these chimeric proteins were cloned into a pSCSTa vector with a C-terminal Fc tag and expressed in 293T cells. Protein expression and purification followed the same procedure as described for the biparatopic binders. Protein sequences were retrieved from the UniProt database. For clarity and consistency, the HER2-ECD sequence was numbered according to the UniProt entry (P04626).

### 2.3 Viability assays

For ligand-independent assays, tumor cells (approximately 2 × 10^3^ cells per well) were seeded into 96-well plates overnight at 37°C with 5% CO_2_. Serially diluted antibodies (1:5 dilution series) were added and incubated with cells for 5 days. Antibody-mediated growth inhibition was evaluated using Cell Counting Kit-8 (CCK-8, Dojindo) according to the manufacturer’s instructions. Briefly, the culture supernatant was removed, and cells were incubated with fresh medium containing 10% (v/v) CCK-8 reagent at 37°C/5% CO_2_ for 2 h. Absorbance at 450 nm was measured using a BioTek plate reader. For ligand-dependent assays, tumor cells were stimulated with either 5 nM EGF (SinoBiological, GMP-10605-HNAE) or 1 nM HRG (SinoBiological, 11609-HNCH). Percent viability was calculated relative to untreated controls. Data was analyzed by nonlinear regression using the “log (agonist) vs. response-variable slope (four parameters)” model in GraphPad v10.2.0. One-way ANOVA was performed to compare the inhibitory effects of different antibodies at 150 nM or 75 nM, and the corresponding p-values were determined.

### 2.4 Western blot analysis

NCI-N87 cells were seeded in Nunc 6-well multi-dishes at a density of approximately 1.2 × 10^6^ cells per well and incubated overnight at 37°C. The following day, cells were treated with 150 nM of either anti-HER2 single agent, negative control antibodies, or T + P (150 nM each) for 24 h at 37°C. After treatment, cells were washed twice with pre-cooled PBS buffer and lysed in NP-40 lysis buffer (Solarbio, N8032). Lysates were centrifuged at 4°C for 5 mins, and the supernatant was collected. Protein concentrations were determined using a BCA protein assay kit (Solarbio, PC0020). Equal amounts of proteins were separated on 10% SDS-PAGE gels in Tris-Glycine running buffer (Solarbio, T1070) and transferred to polyvinylidene fluoride (PVDF) membrane at 350 mA for 90 mins using transfer buffer (Solarbio, D1061). Membranes were blocked with 5% non-fat dry milk (NFDM) blocking buffer for 1 h at room temperature and then incubated with primary antibodies against HER2 (rabbit mAb, CST, 2165) or GAPDH (mouse mAb, Solarbio, K000026M) for 1 h at room temperature. The membrane blots were then washed and incubated with HRP-conjugated goat anti-rabbit IgG (Solarbio, SE134) or goat anti-mouse IgG (Solarbio, SE131). Washes were performed using TBS-T buffer (25 mM Tris-HCl, pH 8, 137 mM NaCl, 2.7 mM KCl, and 0.05% Tween-20). Protein bands were imaged using a ChemiDoc XRS+ Gel imaging system (Bio-Rad) and quantified with ImageJ software.

### 2.5 High-performance size-exclusion chromatography

The homogeneity of the HER2-targeted binder was assessed by HPSEC using an Agilent 1200 series system (Agilent Technologies, Santa Clara, CA) equipped with a TSK Gel G 3000 pwxl (7.8 × 300 mm; TOSOH, Tokyo, Japan) equilibrated in PBS buffer. The flow rate was maintained at 0.5 mL min^-1^, and protein elution was monitored by absorbance at 280 nm.

### 2.6 Enzyme-linked immunosorbent assay

To identify the binding epitopes of biparatopic binders, 96-well plates were coated with either wild-type or chimeric HER2-ECD proteins in PBS at 4°C overnight. Plates were then blocked with 2% NFDM at room temperature for 2 h. Serial dilutions of biparatopic binders or other anti-HER2 antibodies were added and incubated at room temperature for 1 h. HRP-conjugated mouse anti-human IgG Fc antibody (GenScript, A01854) was applied for detection and incubated for 30 mins at room temperature. The supernatant was removed, and TMB substrate was added for the color reaction. The reaction was stopped with 1M H_3_PO_4_, and the absorbance at 450 nm was measured using a BioTek plate reader. ELISA data were analyzed using GraphPad Prism v10.2.0. Curve fitting was performed by nonlinear regression using the “log (agonist) vs. response-variable slope (four parameters)” model to determine the EC_50_ value.

### 2.7 Cell-surface binding by flow cytometry

HER2-expressing tumor cells were resuspended in PBS supplemented with 2% FBS and seeded into 96-well plates at 6 × 10^4^ cells per well. The cells were treated with diluted anti-HER2 agents on ice for 1 h. Following incubation, unbound agents were removed by washing, and cells were subsequently incubated with PE-conjugated anti-human IgG Fc secondary antibody (Abcam, 98596; 1:1000) at 4°C for 30 mins. After washing, cells were resuspended in 120 μL PBS containing 2% FBS. Mean fluorescent intensity (MFI) was acquired using a Beckman Coulter flow cytometer. Binding data was analyzed using GraphPad Prism v10.2.0.

### 2.8 Internalization assay

Receptor internalization was assessed by flow cytometry and confocal microscopy. A flow cytometry assay was performed as previously described (32). Briefly, HER2-expressing tumor cells were treated with HER2-targeting agents on ice for 1 h. After washing with PBS + 2% FBS, one aliquot of cells was maintained on ice, while the remaining cells were incubated at 37°C for 4 h. Cells were fixed with cold 4% paraformaldehyde (PFA) for 20 mins, stained with PE-conjugated anti-human IgG Fc secondary antibody (Abcam, 98596; 1:1000 dilution) at 4°C for 30 mins, and analyzed by Beckman Coulter flow cytometer. The receptor internalization was calculated as the percentage loss of MFI at 37°C relative to that measured on ice.

For the confocal imaging assay, NCI-N87 or MCF-7 cells were seeded onto glass coverslips and cultured overnight at 37°C with 5% CO_2_. Cells were incubated with 75 nM anti-HER2 antibodies or negative control antibodies at 4°C for 1 h. After washing to remove unbound antibodies, cells were incubated at 37°C for 4 h, followed by fixation with 4% PFA for 20 mins at room temperature. Permeabilization was performed using Triton X-100. Cells were blocked with PBS containing 10% goat serum (Solarbio, SL038). To detect internalized antibody-receptor complexes, cells were stained with goat anti-human IgG Fc antibody conjugated to DyLight^®^ 488 (green; Abcam, ab98619). Lysosomes were stained using anti-LAMP1 antibody (Abcam, ab25630), followed by Alexa-Fluor 647-labeled goat anti-mouse IgG H&L (magenta; Abcam, ab150115). Nuclear staining was performed using DAPI (blue), and coverslips were treated with an antifade mounting medium (Beyotime, P0126). Imaging was carried out using a Nikon A1 confocal microscope and analyzed with NIS-Elements Viewer software. Fluorescence intensity from magenta, green, and blue channels was quantified using ImageJ. Average intensity values were analyzed using GraphPad Prism v10.2.0.

### 2.9 Structure analysis

The structures of nanobody-HER2 complexes were predicted using Alphafold 3 (https://alphafoldserver.com/), following the server’s guidelines (41). Briefly, the amino acid sequences of the anti-HER2 nanobodies and HER2-ECD were input into Alphafold 3, and the prediction was performed using default parameters. The predicted structures were visualized and analyzed by Chimera and PyMOL. The PISA server (www.ebi.ac.uk/pdbe/pisa) was used to analyze the nanobody-HER2 interactions.

### 2.10 Statistical analysis

Statistical analysis was performed using GraphPad Prism v10.2.0. Specific tests are described in the relevant method (see above) or figure legends. Quantitative data were analyzed using one-way ANOVA or 2-way ANOVA, as appropriate. Significance thresholds were defined as follows: p < 0.0332 (*), p < 0.0021 (**), p < 0.0002 (***), and p < 0.0001 (****).

## 3 Results

### 3.1 Construction of the biparatopic anti-HER2 nanobody binder

**Figure 1:**
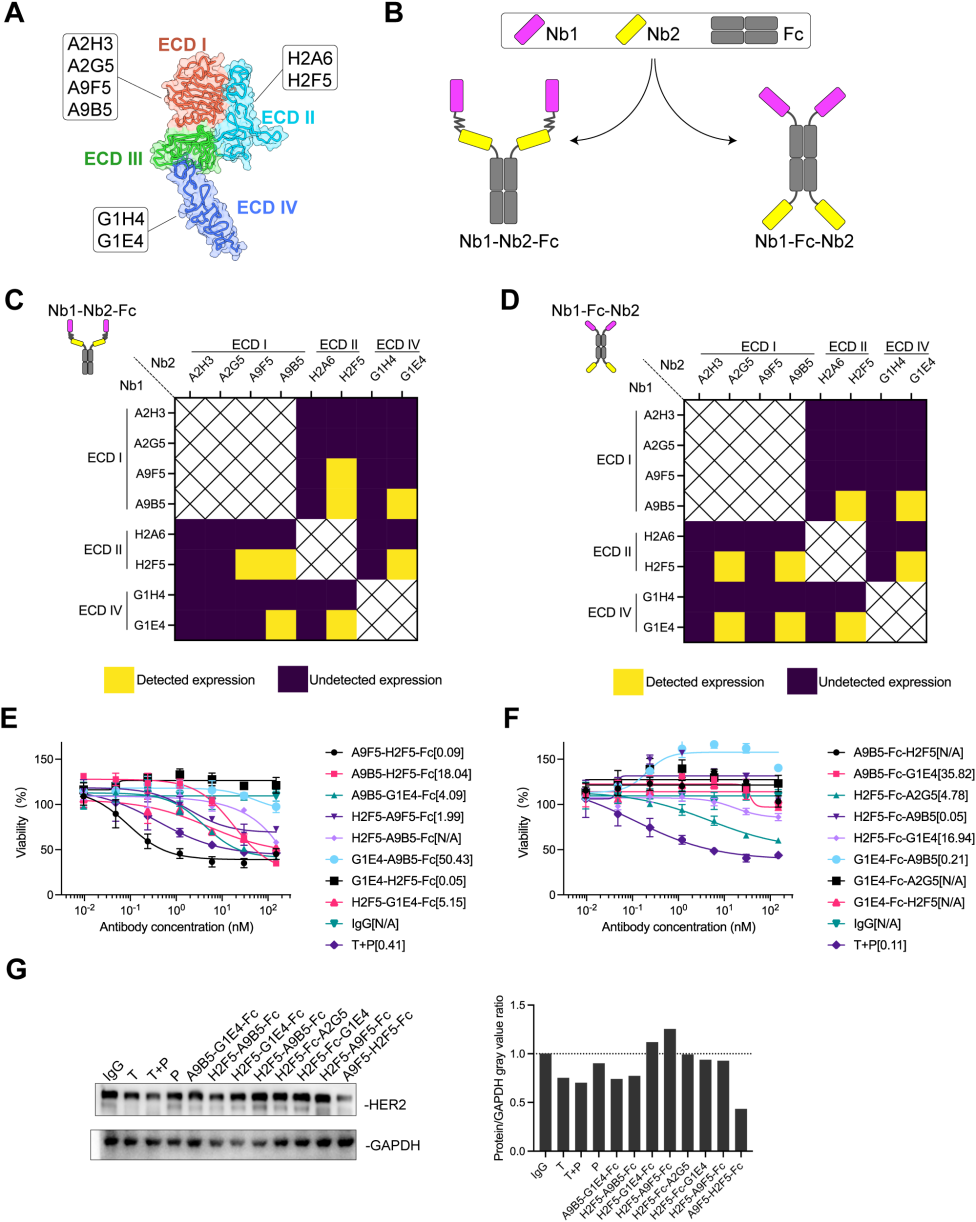
AH is a biparatopic HER2-targeted binder that promotes HER2 downregulation and mediates growth inhibition in HER2-expressing cancer cells. **(A)** Schematic representation of the binding epitopes of high-affinity HER2-specific nanobodies. The crystal structure of HER2-ECD monomer (ID: 1N8Z) was retrieved from the Protein Data Bank (PDB). **(B)** Structural design of HER2-targeting biparatopic binders. In the first format (Nb1-Nb2-Fc), two distinct nanobodies were fused in tandem to the N-terminus of human IgG Fc via flexible (GGGGS)_3_ linkers. In the second format (Nb1-Fc-Nb2), nanobodies were individually fused to the N- and C-terminus of the Fc domain. **(C, D)** Expression of biparatopic binders in Expi293 cells. Binders with detectable expression were shown in yellow; those with undetectable expression were shown in deep purple. **(E, F)** Growth inhibition of NCI-N87 cells by Nb1-Nb2-Fc **(E)** and Nb1-Fc-Nb2 **(F)**. The EC_50_ values (nM) were indicated. Data are mean ± SD from n = 3. **(G)** HER2 downregulation induced by HER2-targeting binders. Immunoblots (left) show HER2 levels in NCI-N87 cells treated with 150 nM anti-HER2 agents. Quantification (right) showed protein/GAPDH gray value ratios determined by ImageJ software. Source data are available in the Source Data file.

Combinations of antibodies and biparatopic antibodies (bpAbs) provide unique MOAs and therapeutic benefits that surpass those of canonical monospecific antibodies by targeting non-overlapping epitopes (23, 25, 42, 43). To develop an effective and cost-efficient therapeutic agent for inhibiting the growth of HER2-positive tumors, nanobody-based biparatopic binders were screened. Previously, we reported a series of high-affinity nanobodies targeting distinct epitopes of HER2-ECD (**Figure 1A**). These nanobodies demonstrated potent synergistic antitumor activity in trastuzumab-resistant cancer cells (32). Here, we constructed two tetravalent biparatopic binder formats by fusing anti-HER2 nanobodies recognizing non-overlapping epitopes to human immunoglobulin G1 (IgG1) Fc regions (**Figure 1B**). The first format consisted of two nanobodies fused in tandem to the N-termini of the Fc domain (designated Nb1-Nb2-Fc), whereas the second format involved nanobodies fused to both the N- and C-termini of the Fc domain (designated Nb1-Fc-Nb2). A total of 80 biparatopic binders were generated and expressed in Expi293 cells. Regardless of the format, more than half of the binders exhibited undetectable expression, suggesting certain configurations may interfere with fusion protein expression (**Figures 1C, D**).

Fc-fusion strategies have been reported to enhance nanobody functionality by increasing valency (38, 40, 44, 45). We hypothesized that tetravalent biparatopic binders benefit from avidity, thereby further enhancing antitumor activity. NCI-N87 cells with high HER2 expression level were selected to evaluate growth inhibition mediated by biparatopic binders compared with trastuzumab plus pertuzumab (T + P). Within the Nb1-Nb2-Fc format, A9F5-H2F5-Fc exhibited the greatest inhibitory effect, achieving an EC_50_ value fourfold lower than T + P (**Figure 1E**). No binder in Nb1-Fc-Nb2 format demonstrated superior inhibition compared with T + P (**Figure 1F**). Interestingly, G1E4-Fc-A9B5, composed of nanobodies targeting ECD IV and ECD I, displayed agonistic activity.

In HER2-dependent tumor cells, inducing HER2 downregulation represents a viable strategy for anti-HER2 therapeutics (26, 46). It was next investigated whether tetravalent biparatopic binders induce HER2 receptor downregulation. HER2 protein levels were quantified from NCI-N87 cell lysates following treatment with binders or anti-HER2 antibodies. As shown in **Figure 1G**, A9F5-H2F5-Fc reduced HER2 levels to less than 50% of baseline. Trastuzumab, T +P, A9B5-G1E4-Fc, and H2F5-A9B5-Fc elicited a weaker effect in NCI-N87 cells. These findings indicate that the tetravalent biparatopic binder A9F5-H2F5-Fc induces HER2 downregulation and inhibits the growth of HER2-expressing tumor cells, demonstrating strong developability and manufacturability. Accordingly, A9F5-H2F5-Fc, hereafter referred to as AH, was selected for further experiments.

### 3.2 Dual targeting of HER2 ECD I and ECD II achieves high binding saturation

**Figure 2:**
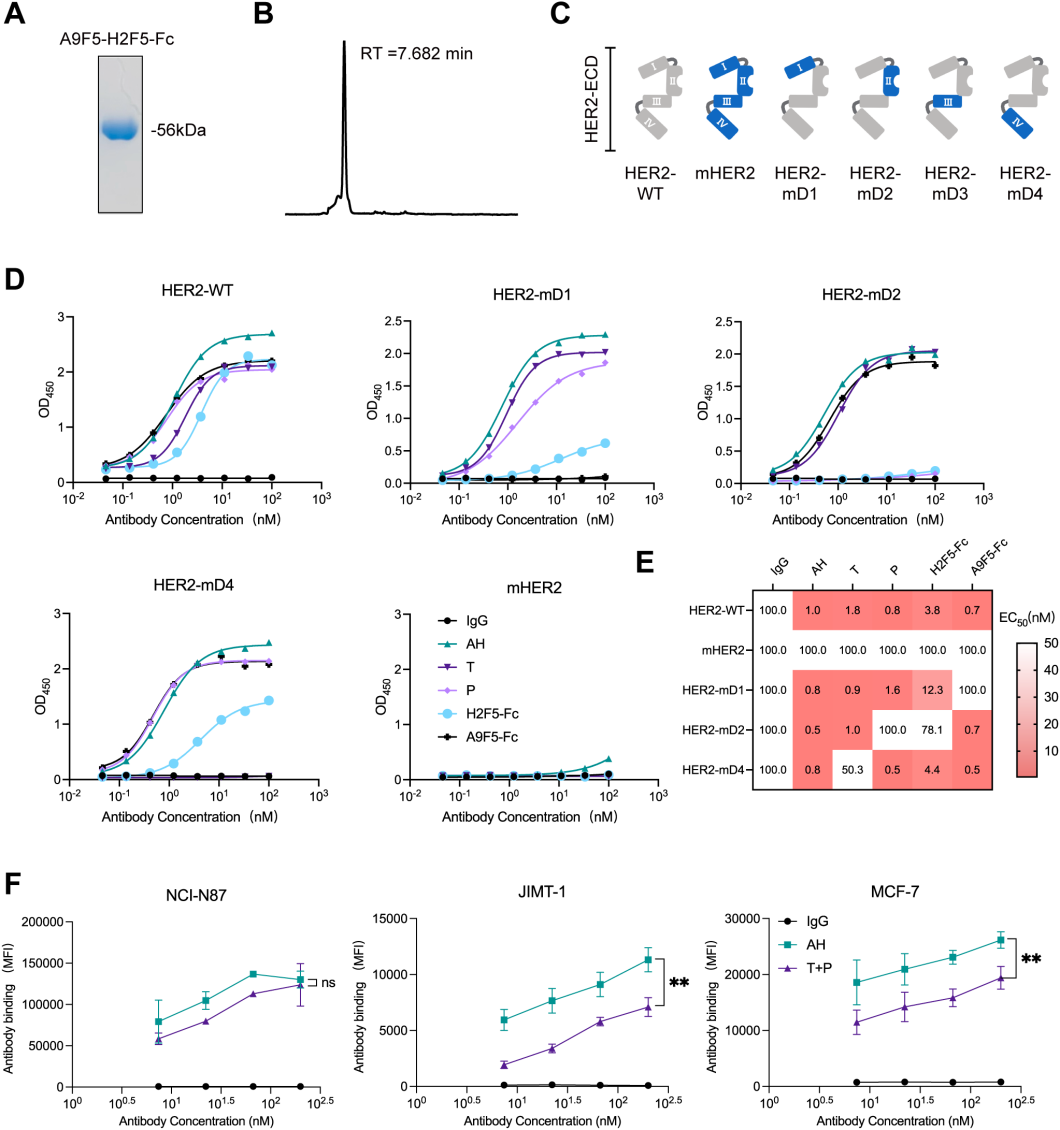
Dual epitope targeting by AH confers high HER2 binding saturation. **(A)** Coomassie-stained SDS-PAGE gel showing high purity of purified AH. **(B)** HPSEC profile of AH demonstrating molecular homogeneity. The retention time (RT) was indicated. **(C)** Schematic representation of chimeric HER2-ECD constructs. In HER2-mD1 to HER2-mD4, individual domains of human HER2 (ECD I: T23-R217; ECD II: T218-C342; ECD III: Y344-A510; ECD IV: C511-T652) were replaced with the corresponding regions from the murine homolog. **(D)** ELISA analysis of AH binding to wild-type and chimeric HER2-ECD proteins. SDS-PAGE and HPSEC characterization data for all anti-HER2 agents shown in **(D)** are provided in the Source Data file. **(E)** Binding epitope mapping of the AH based on EC_50_ values (nM), derived from ELISA, displayed as a heat map. EC_50_ values are indicated within each cell, with values >100 nM uniformly shown as 100 nM. The color gradient represents binding affinity, with lower EC_50_ values depicted in red. **(F)** Flow cytometry analysis of AH binding in trastuzumab-sensitive and -resistant HER2-positive cell lines. Cells were treated with AH or trastuzumab plus pertuzumab (T + P). Data are mean ± SD from n = 3. Statistical significance was assessed using unpaired 2way-ANOVA: p < 0.0332 (*), p < 0.0021 (**), p < 0.0002 (***), and p < 0.0001 (****). The p-values were provided in **Supplementary Table 1**. Source data are available in the Source Data file.

We next characterized the binding properties of the nanobody−based biparatopic binder AH. Purified AH exhibited > 95% purity by SDS–PAGE and migrated at the expected molecular weight (~56 kDa) (**Figure 2A**). Analytical size−exclusion chromatography revealed a single dominant peak with negligible aggregation, indicating biochemical homogeneity (**Figure 2B**). To map the binding epitopes of AH, a panel of recombinant chimeric HER2-ECD proteins was generated, in which each human subdomain (ECD I–IV) was individually replaced by its murine counterpart (**Figure 2C**). As HER2-mD3 expression was undetectable, only HER2-mD1, HER2-mD2, and HER2-mD4 were included in ELISA analysis. The lack of detectable HER2-mD3 expression is likely due to structural perturbations caused by the non-natural domain swap, which may interfere with proper protein folding in mammalian cells. AH bound robustly to wild−type human HER2 and all three chimeric HER2-ECD proteins, while showing negligible binding to murine HER2 (**Figure 2D**). The parental nanobodies A9F5 and H2F5 were sensitive to substitutions in ECD I and ECD II, respectively. Control antibodies behaved as expected—trastuzumab was sensitive to ECD IV substitution and pertuzumab to ECD II—validating the domain−swap strategy. ELISA−derived EC_50_ values confirmed these patterns: domain substitution had no effect on AH binding, and AH maintained low EC_50_ values (≤ 1 nM) for both wild−type and chimeric HER2-ECD proteins (**Figure 2E**). These data indicate that AH simultaneously engages HER2 ECD I and ECD II.

Cell-surface binding saturation of AH was compared with that of the clinical antibody combination trastuzumab plus pertuzumab (T + P) across HER2-positive tumor models. In NCI-N87 cells with high HER2 expression, AH achieved saturation comparable to T + P, whereas in trastuzumab-resistant lines (MCF7 and JIMT-1), AH reached ~1.3–l.6-fold higher maximal binding than T + P (**Figure 2F**, **Supplementary Table 1**). Together, these results demonstrated that AH retains the binding features of parent nanobodies A9F5 and H2F5, enabling co-engagement of non-overlapping epitopes on ECD I and ECD II and achieving high binding saturation.

### 3.3 Enhanced HER2 internalization induced by AH alone or with trastuzumab

**Figure 3:**
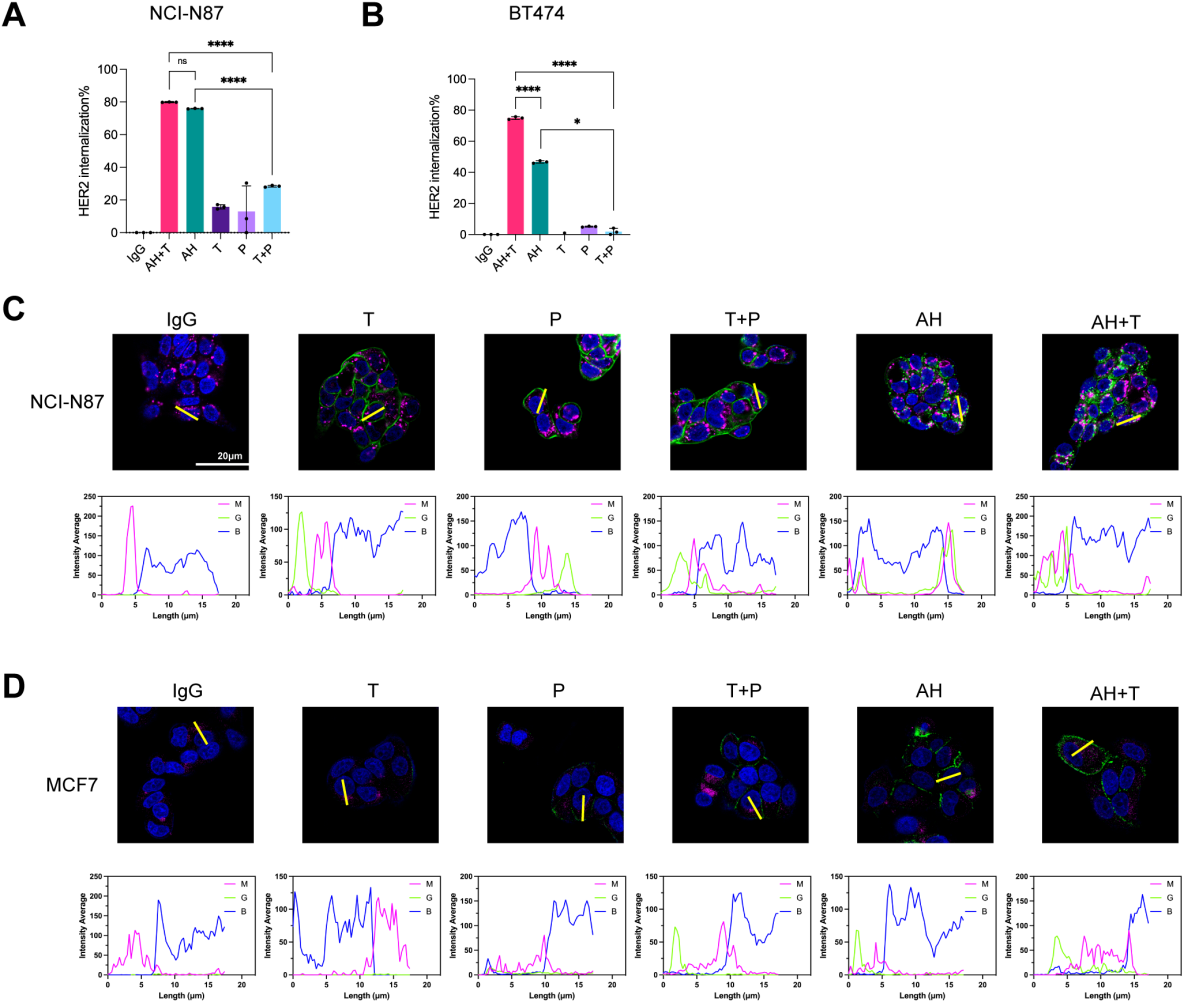
AH promotes HER2 internalization alone and in combination with trastuzumab in HER2-positive cancer cells. **(A, B)** HER2 internalization in tumor cells following treatment with AH alone or in combination with trastuzumab. Internalization was assessed in NCI-N87 **(A)** and BT474 **(B)** cells. The receptor internalization was calculated as the percentage loss of MFI at 37°C relative to that measured on ice. Data are mean ± SD from n = 3. Statistical significance was determined by one-way ANOVA: p < 0.0332 (*), p < 0.0021 (**), p < 0.0002 (***), and p < 0.0001 (****). P-values were provided in **Supplementary Table 2**. **(C, D)** Confocal imaging of antibody-HER2 complexes in NCI-N87 **(C)** and MCF-7 **(D)** cells. Antibody-HER2 complexes were labeled with DyLight^®^ 488-labeled antibody (green), lysosomes with AF647-labeled antibody (magenta), and nuclei with DAPI (blue). Regions of interest (ROIs) were selected to include representative cells with clear membrane and cytoplasmic signals while avoiding overlapping cells or artifacts, ensuring unbiased quantification. Scale bars, 20 μm. Fluorescence intensity from magenta (M), green (G), and blue (B) channels was quantified using ImageJ to assess colocalization of immunocomplexes with lysosomes. Source data are available in the Source Data file.

BpAbs are theoretically capable of cross-linking cell-surface receptors into higher-order clusters because of multivalent engagement of two non-overlapping epitopes (43, 47, 48). Subsequently, the ability of the biparatopic binder AH to promote HER2 internalization was evaluated. Given that AH targets non-competing epitopes relative to trastuzumab, the potential for synergistic activity upon co-administration was also investigated. Internalization was quantified by flow cytometry using the on-ice versus 37°C assay described in Methods. In NCI-N87 cells, trastuzumab, pertuzumab, and T + P induced limited internalization (< 30%), whereas AH and AH + T yielded high-level internalization (> 75%) (**Figure 3A**, **Supplementary Table 2**). The internalization levels elicited by AH and AH+T were comparable (**Figure 3A**, **Supplementary Table 2**). In BT474 cells, trastuzumab, pertuzumab, and their combination (T + P) elicited minimal internalization, whereas AH triggered a pronounced increase. Co-treatment with trastuzumab and AH (AH+T) further enhanced internalization relative to AH alone (**Figure 3B**, **Supplementary Table 2**).

To visualize intracellular trafficking, we used confocal microscopy to track HER2-binder internalization and lysosomal trafficking in two cell lines. In NCI-N87 assays, AH alone and AH + T each induced robust HER2 internalization, and the internalized AH-HER2 complexes co-localized with lysosomes (**Figure 3C**). By contrast, trastuzumab, pertuzumab, and T + P remained largely at the cell surface. In MCF-7 (low HER2 expression) cells, trastuzumab, pertuzumab, and T + P showed limited surface binding with no detectable internalization. AH alone and AH + T showed increased cell-surface binding but only a weak intracellular staining (**Figure 3D**). These data indicated that the AH-driven internalization (alone or with trastuzumab) depends on the level of HER2 expression. In summary, AH drives robust HER2 internalization and lysosomal trafficking, and co-treatment with trastuzumab amplifies this response.

### 3.4 Synergistic growth inhibition in trastuzumab-resistant HER2-positive cells

**Figure 4:**
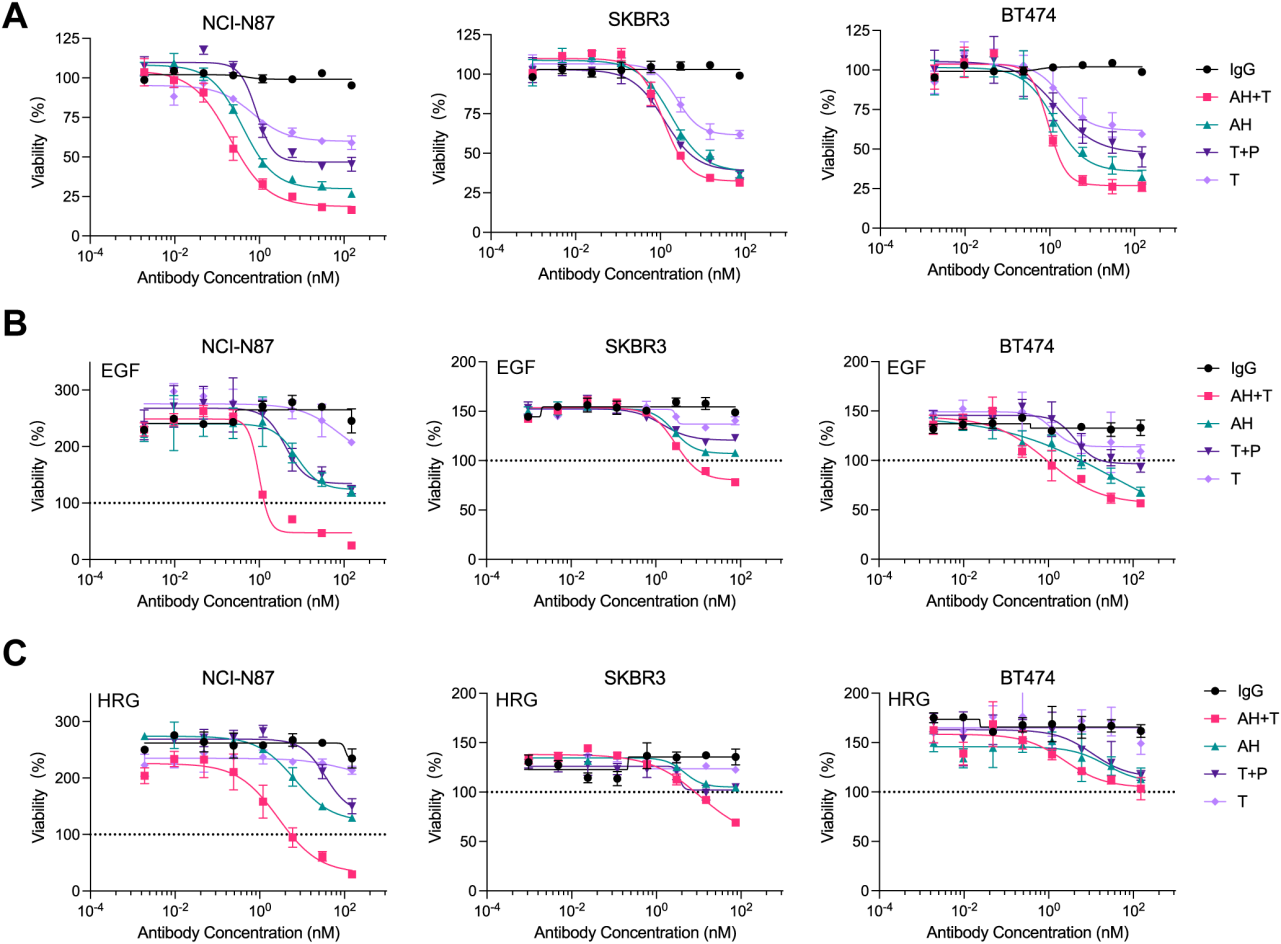
AH synergizes with trastuzumab to inhibit the proliferation of ligand-independent and ligand-dependent HER2-expressing cancer cells. **(A)** Ligand-independent growth inhibition induced by AH alone or in combination with trastuzumab (AH + T) in NCI-N87, SKBR3, and BT474 cells. **(B)** Growth inhibition under EGF-stimulation following treatment with AH or AH + T. **(C)** Growth inhibition under HRG-stimulation following treatment with AH or AH + T. Serum-starved tumor cells were incubated in the presence of 1 nM HRG or 5 nM EGF. Percent viability was calculated relative to untreated controls. Horizontal dotted line (black) represents the viability of non-treated cells referenced to 100%. Data represent mean ± SD (n = 3). Statistical significance was assessed using one-way ANOVA: p < 0.0332 (*), p < 0.0021 (**), p < 0.0002 (***), and p < 0.0001 (****). The p-values for comparisons of percent viability at 150 nM or 75 nM treatment were provided in **Supplementary Table 3**. Source data are available in the Source Data file.

Failure of trastuzumab therapy can arise from ligand-mediated compensatory activation of other HER family receptors (49–51). We therefore asked whether the biparatopic binder AH, alone or combined with trastuzumab (AH + T), suppresses cell growth in the absence or presence of exogenous ligand across three HER-expressing cell lines (NCI-N87, SKBR3, and BT474 cells). In ligand-independent assays, AH and AH + T each induced significantly greater growth inhibition than trastuzumab in all three cell lines, and were comparable or more potent than the antibody combination (T + P) (**Figure 4A**, **Supplementary Table 3**). Adding trastuzumab measurably increased AH activity under ligand−free conditions in NCI-N87 cells, yet AH+T conferred no additional benefit over AH alone in SKBR3 and BT474 cells.

The dual HER2 inhibition with T + P has been reported to achieve more comprehensive blockade of HER2 signaling and improved therapeutic outcome in trastuzumab-resistant tumors (12, 13, 52, 53). We next compared AH and AH + T under ligand-stimulating conditions using epidermal growth factor (EGF) or heregulin (HRG). As expected, trastuzumab showed minimal activity in the presence of EGF or HRG, whereas T + P was more effective (**Figures 4B, C**). AH surpassed T + P in EGF-stimulated SKBR3 and BT474 cells (**Figure 4B**, **Supplementary Table 3**). AH + T delivered stronger ligand-dependent growth inhibition than T + P in all three cell lines, except that activity was comparable between the two in HRG-stimulated BT474 cells (**Figures 4B, C**). AH + T also outperformed AH alone in ligand-stimulated NCI-N87 and SKBR3 cells, while the two were similar in BT474 cells (**Figures 4B, C**). Collectively, AH alone achieved growth inhibition that was equal to or greater than T + P regardless of ligand status, and combining AH with trastuzumab further enhanced activity in several ligand−driven, trastuzumab−resistant contexts. These findings support a functional synergy between AH and trastuzumab in HER2−positive cancer models.

### 3.5 Structural modeling suggests a *trans*-binding mechanism underlying AH activity

**Figure 5:**
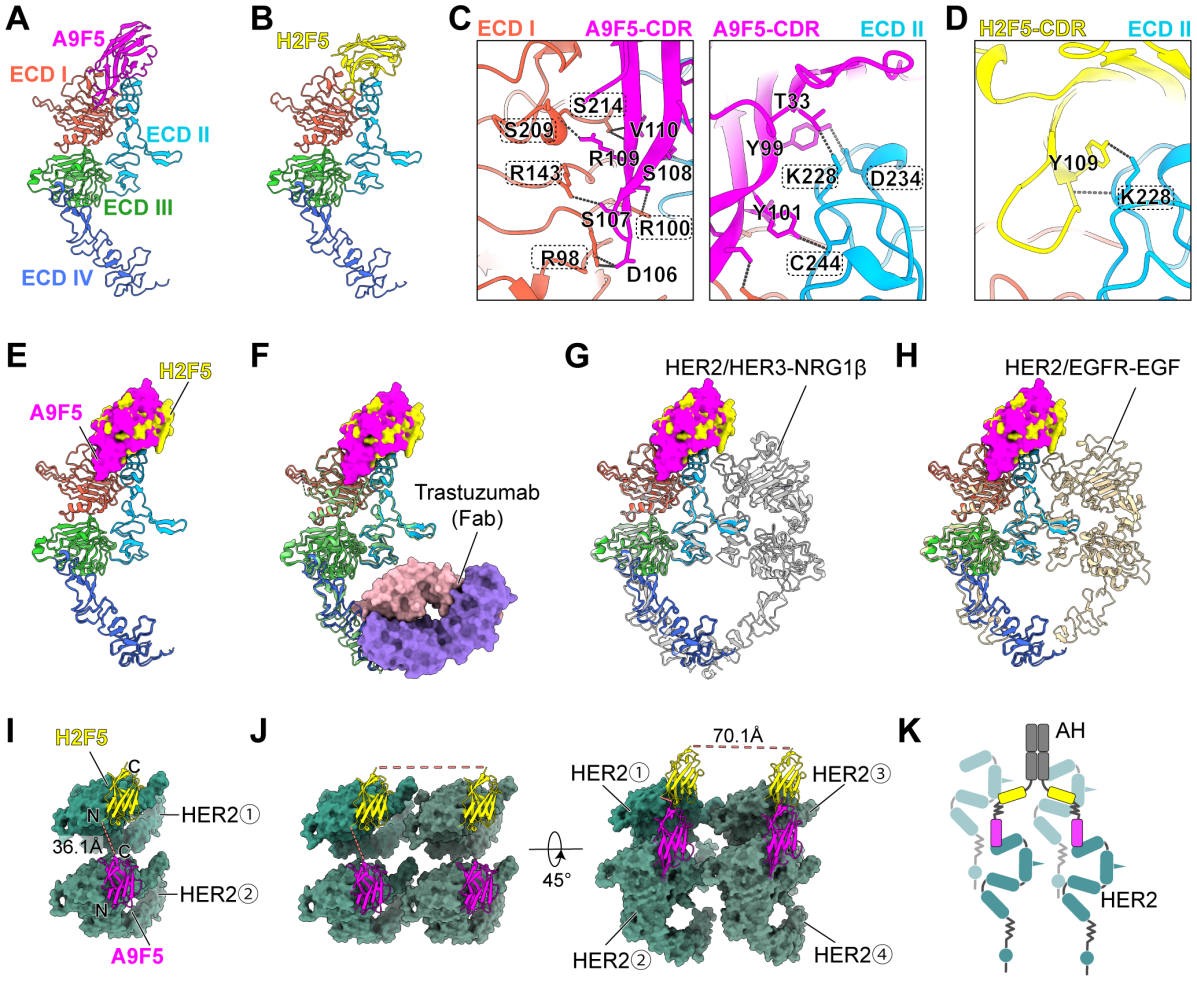
Structural investigation suggests a *trans*-binding mode of AH to HER2-ECD. **(A, B)** Predicted structures of A9F5-HER2 complex **(A)** and H2F5-HER2 complex **(B)** generated by Alphafold 3. Nanobodies A9F5 and H2F5 were shown in magenta and lemon yellow, respectively. HER2 ECD I-IV are colored tomato, deep sky blue, lime green, and royal blue, respectively. The predicted structures are available in the Source Data file. **(C, D)** Predicted interactions between A9F5 **(C)** or H2F5 **(D)** and HER2-ECD. Dashed lines represented interatomic distances < 4 Å The detailed interaction sites were provided in **Supplementary Table 4**. **(E)** Structural superposition of A9F5-HER2 and H2F5-HER2 complexes showing steric hindrance, suggesting that both nanobodies cannot bind to a single HER2 molecule simultaneously. **(F)** Superposition of A9F5-HER2, H2F5-HER2, and the trastuzuamb-HER2 complex (PDB ID: 1N8Z). Trastuzumab Fab was shown in medium purple (light chain) and light pink (heavy chain). **(G)** Superposition of A9F5-HER2, H2F5-HER2, and the HER2-HER3-NRG1β dimer complex (PDB ID: 7MN5). The HER2-HER3-NRG1β dimer complex was shown in light gray. **(H)** Superposition of A9F5-HER2, H2F5-HER2, and the HER2-EGFR-EGF dimer complex (PDB ID: 8HGO). The HER2-EGFR-EGF dimer complex was shown in wheat. **(I)** Putative *trans*-binding model of AH engaging adjacent two HER2 molecules via a single nanobody arm. HER2-ECD was colored sea green. Dashed lines (watermelon pink) indicate distances between the C-terminus of A9F5 and the N-terminus of H2F5. **(J)** Putative binding model of AH engaging four HER2 molecules via dual nanobody arms. The distances between the C-termini of H2F5 domains from different arms were indicated by dashed lines (watermelon pink). **(K)** Schematic illustration of AH-driven HER2 clustering on the cell surface through multivalent *trans*-binding mode.

To define the structural basis of AH engagement, complexes between each nanobody and HER2-ECD were predicted using AlphaFold 3. The top−ranked models placed A9F5 across ECD I and ECD II, and H2F5 on ECD II (**Figures 5A, B**). Interface residues were identified using the PISA server. A9F5 formed eight hydrogen bonding contacts, including five contacts with ECD I (R98, R100, R143, S209, and S214) and three contacts with ECD II (K228, D234, and C244) (**Figure 5C**, **Supplementary Table 4**). H2F5 engaged two hydrogen bonding contacts, both involving K228 (**Figure 5D**, **Supplementary Table 4**). Although both nanobodies interact with K228, their overall binding surfaces are spatially distinct. A9F5 spans ECD I and ECD II, whereas H2F5 is confined to ECD II, indicating minimal epitope overlap. Notably, while modeling suggested that A9F5 interacts with both ECD I and ECD II and that H2F5 engages ECD II through K228, ELISA assays indicated predominant binding of A9F5 to ECD I and a loss of H2F5 reactivity against HER2-mD2. These discrepancies likely reflect conformational perturbations introduced by the chimeric domain-swapped proteins, which may compromise proper epitope presentation despite residue conservation.

Superposition of the A9F5-HER2 and H2F5-HER2 complexes revealed substantial steric interference between the nanobody variable domains (**Figure 5E**). The relative approach angles bring the frameworks and complementarity-determining regions (CDR) loops into proximity, generating clashes that would preclude simultaneous *cis*-binding to a single HER2 molecule. Although minor conformational adjustments could partially alleviate overlap, the geometry suggests that AH is predisposed to engage HER2 in a *trans*-configuration.

To assess compatibility with clinically and biologically relevant HER2 states, the predicted complexes were superposed onto structures of trastuzumab−bound HER2 (PDB ID: 1N8Z), the HER2-HER3-NRG1β dimer (PDB ID: 7MN5), and the HER2-EGFR-EGF dimer (PDB ID: 8HGO) (**Figures 5F–H**). No major steric clashes were observed between the modeled A9F5 or H2F5 poses and trastuzumab, consistent with the functional synergy between AH and trastuzumab. Epitope accessibility was largely retained in the context of HER2-driven heterodimers, although proximity to the dimerization arms could modulate binding orientation or avidity (**Figures 5G, H**). These comparisons indicate that AH could recognize HER2 across multiple receptor conformational states present on tumor cells.

A9F5 and H2F5 are linked in tandem by a flexible (GGGGS)_3_ linker (~57 Å) within AH. Structural alignment of the nanobody-HER2 complexes showed that the distance between the A9F5 C−terminus and H2F5 N−terminus, when bound to adjacent HER2 molecules, is ~36.1 Å—well within the reach of the (GGGGS)_3_ linker (**Figure 5I**). This geometry would permit a single AH arm to bridge two HER2 molecules in *trans*-mode without requiring full linker extension, providing a structural basis for receptor clustering on the cell surface. Full−length AH incorporates an IgG Fc, yielding a tetravalent (two−arm) architecture. Modeling of the Fc−linked dimer indicated that the distance between the two biparatopic arms is ~70.1 Å (**Figure 5J**). This inter−arm span is shorter than the mean distance (117–134 Å) between antigen−binding sites in conventional IgG. This compact arrangement may draw additional HER2 molecules into proximity. Consequently, a single AH molecule could, in principle, engage up to four HER2 molecules (two per arm), leading to the formation of higher-order oligomers on the cell surface (**Figure 5J**). These structural analyses support a model in which AH promotes multivalent HER2 clustering through a *trans*-binding mechanism (**Figure 5K**).

## 4 Discussion

BpAbs represent a promising strategy to improve HER2-targeted therapy by simultaneously engaging multiple epitopes on the HER2-ECD, thereby enhancing receptor clustering, internalization, and inhibition of downstream signaling (54). In this work, a biparatopic nanobody-based binder, A9F5-H2F5-Fc (AH), was designed to target non-overlapping epitopes on ECD I and ECD II. AH exhibited potent and selective antitumor activity in HER2-overexpressing tumor models, including trastuzumab-resistant cell lines, underscoring its potential as a next-generation HER2-targeted agent.

Activating or acquired mutations within HER2-ECD have emerged as key drivers of antibody resistance (55–57). Such mutations can reduce the binding affinity of existing anti-HER2 antibodies and diminish therapeutic efficacy (26). Biparatopic constructs may retain sufficient binding affinity even in the presence of HER2 mutations, preserving antitumor activity (**Figure 2E**). Notably, the development of bsAbs requires biologically informed design to select epitope locations, affinity ranges, and optimal formats (58–61). The design of AH was guided by complementary epitope recognition between A9F5 and H2F5. Structural modeling revealed that A9F5 spans ECD I and ECD II, whereas H2F5 binds a distinct region within ECD II, resulting in minimal epitope overlap. This configuration enables cooperative *trans*-binding and receptor clustering, which are not achievable with a single VHH-Fc construct. Compared with monospecific formats, the biparatopic architecture enhances avidity and promotes HER2 internalization and degradation, leading to superior functional efficacy. These findings highlight the structural and mechanistic rationale underlying the selection of the A9F5-H2F5 combination for bpAb design.

An unbiased screening strategy was employed to identify the optimal AH format. First, we found that some constructs exhibited reduced expression without sharing common features (**Figures 1C,D**). This observation likely reflects structural or steric constraints imposed by certain nanobody combinations, which may interfere with proper folding, secretion, or overall protein stability. Such challenges underscore the importance of empirical screening to identify well-expressed and functional biparatopic constructs. Second, the Nb1-Nb2-Fc format demonstrated superior antitumor activity compared to the Nb1-Fc-Nb2 format (**Figures 1E, F**). This may be due to the Nb1-Fc-Nb2 configuration imposing steric constraints that interfere with simultaneous engagement of both epitopes, thereby reducing effective biparatopic binding. These observations highlight that both linker orientation and nanobody placement are critical for preserving productive dual-epitope binding and maximizing functional activity of biparatopic constructs. Third, ECD-I engagement emerged as a key determinant of functionality. Although ECD I does not directly mediate HER2 dimerization, it contributes to the formation of a binding pocket for the DA of other HER2 partners (42, 62–64). Anti-HER2 bsAbs incorporating ECD-I binding modules have shown promising efficacy in preclinical studies (43, 65). For instance, zenocutuzumab, a HER2/HER3 bsAb generated by using an ECD I-binding mAb (MF3958) and an anti-HER3 ECD III mAb (MF3178), effectively inhibits HRG-driven proliferation through a “dock & block” mechanism (18). Similarly, our AH construct, targeting ECD I and ECD II, exhibited potent inhibitory activity in ligand-driven tumor models via *trans*-binding and multivalent HER2 clustering (**Figure 5K**). Collectively, these findings underscore the unique structural and functional relevance of ECD I in HER2 biology, and highlight its therapeutic potential as a complementary target to ECD II blockade for overcoming resistance to existing HER2 therapies.

Nanobody affinity was not linearly correlated with bpAb efficacy. A9B5 (KD = 7.8 nM) and A9F5 (KD = 19.4 nM) are affinity-matured variants of the same parent nanobody (32). Despite higher affinity, A9B5-H2F5-Fc displayed lower antitumor efficacy than A9F5-H2F5-Fc (AH), suggesting that affinity alone does not predict functional performance (**Figure 1E**). The moderate affinity of A9F5 may favor productive *trans*-binding to adjacent HER2 molecules, promoting receptor clustering without premature dissociation. Consistently, AH treatment induced HER2 internalization and subsequent protein downregulation, indicating efficient trafficking to lysosomes. This multivalent engagement facilitates receptor degradation and enhanced signal attenuation, thereby contributing to the superior antitumor activity observed for AH. These observations highlight that structural configuration, epitope location, and dynamic binding kinetics collectively determine the functional performance of biparatopic constructs.

In comparison to existing anti-HER2 bpAbs such as anbenitamab and zanidatamab, AH offers unique structural and functional advantages. Both anbenitamab and zanidatamab are IgG1-based constructs targeting HER2 ECD II and ECD IV (23, 24, 66). These bpAbs have demonstrated superior antitumor activity over trastuzumab and pertuzumab by leveraging dual-epitope binding to promote receptor downregulation, signal inhibition, and immune effector functions such as ADCC and complement-dependent cytotoxicity (CDC) (67, 68). However, they require complex engineering platforms—Fc-based heterodimerization for anbenitamab and asymmetric pairing for zanidatamab—resulting in challenges related to heavy chain pairing, manufacturing scalability, and product homogeneity. In contrast, AH employs a tandem VHH-Fc architecture composed of a single polypeptide chain, simplifying expression and minimizing risks of chain mispairing or undesired self-association (**Figure 1B**). This symmetric design ensures consistent product quality and favorable developability (**Figures 2A, B**).

Receptor internalization is a key mechanism through which anti-HER2 agents suppress oncogenic signaling (47, 69, 70). AH was shown to induce significantly higher HER2 internalization rate than trastuzumab, pertuzumab, or their combination in both high- and low-HER2–expressing cell lines (**Figure 3**). Confocal imaging confirmed that AH facilitated HER2 redistribution to intracellular compartments and enhanced lysosomal co-localization (**Figure 3C**). The magnitude of this effect correlated with HER2 expression level and was further potentiated by co-treatment with trastuzumab, consistent with their non-competing epitopes (**Figure 3D**). This enhanced internalization is expected to not only reduce HER2 surface density but also improve ADC delivery, thereby broadening AH’s translational utility.

Ligand-induced HER2 heterodimerization drives compensatory signaling and contributes to trastuzumab resistance (49, 51, 71, 72). Interfering with this ligand-mediated dimerization is therefore a rational strategy to restore the antitumor activity in resistant tumors. The H2F5 nanobody module within AH targets an epitope overlapping with that of pertuzumab (ECD II), suggesting that AH may exert comparable inhibitory effects (32). Indeed, AH alone demonstrated growth inhibition equal to or exceeding that of the trastuzumab-plus-pertuzumab combination, regardless of ligand stimulation. Furthermore, synergistic activity with trastuzumab was observed in ligand-driven models across HER2-positive gastric (NCI-N87) and breast cancer (SKBR3 and BT474) cell lines (**Figure 4**). In ligand-stimulated conditions, the AH-trastuzumab combination outperformed the trastuzumab-pertuzumab combination, indicating the ability of AH as a favorable synergistic partner to overcome ligand-mediated resistance mechanisms (**Figures 4B, C**). The observed synergy between AH and trastuzumab likely arises from complementary mechanisms of HER2 inhibition rather than direct molecular cooperation. Structurally, AH and trastuzumab recognize non-overlapping epitopes on HER2, enabling simultaneous receptor engagement and more complete inhibition of receptor signaling. Functionally, AH promotes HER2 internalization and degradation, while trastuzumab blocks ligand-independent HER2 activation and recruits immune effector mechanisms via its Fc domain. Since AH also contains a human IgG1 Fc fragment, it may similarly engage immune effector pathways such as ADCC and CDC, which are known to contribute to the therapeutic efficacy of IgG1-based antibodies. Although the present study primarily focuses on receptor-level mechanisms, these Fc-mediated immune functions could further potentiate the antitumor activity of AH *in vivo* and merit future investigation. Their combination therefore exerts dual blockade on HER2 signaling—reducing receptor density at the cell surface and suppressing downstream proliferative pathways—leading to enhanced growth inhibition even in trastuzumab-resistant models. Consistent with this, increased receptor internalization was observed upon co-treatment, supporting a synergistic mode of action rather than simple additive effects.

To define the structural basis of AH engagement, complexes between each nanobody and HER2-ECD were predicted using AlphaFold 3. The top-ranked models positioned A9F5 across both ECD I and ECD II, forming eight hydrogen-bonding contacts—including five contacts with ECD I (R98, R100, R143, S209, and S214) and three contacts with ECD II (K228, D234, and C244) (**Figure 5C**, **Supplementary Table 4**)—whereas H2F5 engaged two contacts in ECD II (both involving K228) (**Figure 5D**). These results suggest largely non-overlapping epitopes for A9F5 and H2F5, with K228 as the only shared contact site. Interestingly, experimental mapping by ELISA revealed predominant binding of A9F5 to ECD I and a loss of H2F5 reactivity toward HER2-mD2, despite structural modeling implicating ECD II contacts. This apparent discrepancy likely reflects limitations of the chimeric domain-swap approach: although residues such as K228 are conserved, the replacement of entire subdomains can introduce subtle conformational changes that disrupt the local architecture required for nanobody recognition. Thus, while structural modeling delineates potential interaction interfaces across HER2 domains, ELISA captures the functional epitope accessibility in a cellularly expressed context. Reconciling these results, we propose that A9F5 functionally engages HER2 primarily through ECD I, with auxiliary contacts in ECD II, whereas H2F5 recognition of ECD II is sensitive to conformational context. Superposition of the A9F5-HER2 and H2F5-HER2 complexes further indicated steric interference in *cis*-binding, favoring a *trans*-configuration in which the flexible (GGGGS)_3_ linker (~57 Å) accommodates inter-epitope distances (~36 Å) between adjacent HER2 molecules, and Fc-mediated dimerization enables tetravalent engagement conducive to multivalent receptor clustering (**Figures 5E–K**). This integrated model explains the enhanced receptor internalization and antitumor efficacy of AH compared to conventional anti-HER2 antibodies.

Despite these promising findings, this study has several limitations. First, although AH demonstrated enhanced internalization and antitumor activity *in vitro*, *in vivo* validation using patient-derived xenografts (PDXs) or transgenic models that better recapitulate HER2 expression heterogeneity is warranted. Second, head-to-head comparisons with clinically advanced bpAbs such as anbenitamab or zanidatamab were not conducted. Such comparisons will be essential to benchmark efficacy, Fc-mediated immune effector functions, and pharmacokinetic behaviors. Third, the long-term safety and potential immunogenicity of the nanobody-based format require further evaluation in non-human primates. Finally, the predicted nanobody–HER2 interactions were based on AlphaFold structural models. Although AlphaFold provides near-experimental accuracy, definitive structural insights will require validation by cryo-electron microscopy or X-ray crystallography, complemented by subsequent biological experiments.

## 5 Conclusion

In summary, the biparatopic nanobody-based binder AH combines structural simplicity with potent functional activity, leveraging the advantages of nanobody engineering and biparatopic targeting to address key resistance mechanisms in HER2-positive cancers. AH promotes robust HER2 internalization and degradation, while its modular VHH-Fc format supports favorable manufacturability. In *in vitro* models, AH demonstrates synergy with trastuzumab, suggesting potential utility in combination regimens for tumors refractory to current HER2-directed therapies. Future studies will be essential to define its *in vivo* antitumor efficacy, pharmacokinetics, and immunogenicity, and to assess its translational potential within the evolving landscape of HER2-targeted treatment strategies.

## Data Availability

The datasets presented in this study can be found in online repositories. The names of the repository/repositories and accession number(s) can be found in the article/**Supplementary Material**.

## References

[1] Choong GM Cullen GD O’Sullivan CC. Evolving standards of care and new challenges in the management of HER2-positive breast cancer. CA Cancer J Clin. (2020) 70:355–74. doi: 10.3322/caac.21634, PMID: 32813307

[2] Costa RLB Czerniecki BJ. Clinical development of immunotherapies for HER2(+) breast cancer: a review of HER2-directed monoclonal antibodies and beyond. NPJ Breast Cancer. (2020) 6:10. doi: 10.1038/s41523-020-0153-3, PMID: 32195333 PMC7067811

[3] Oh DY Bang YJ. HER2-targeted therapies - a role beyond breast cancer. Nat Rev Clin Oncol. (2020) 17:33–48. doi: 10.1038/s41571-019-0268-3, PMID: 31548601

[4] Ma Q Jiang H Ma L Zhao G Xu Q Guo D. The moonlighting function of glycolytic enzyme enolase-1 promotes choline phospholipid metabolism and tumor cell proliferation. Proc Natl Acad Sci U.S.A. (2023) 120:e2209435120. doi: 10.1073/pnas.2209435120, PMID: 37011206 PMC10104498

[5] Kovacs E Zorn JA Huang Y Barros T Kuriyan J. A structural perspective on the regulation of the epidermal growth factor receptor. Annu Rev Biochem. (2015) 84:739–64. doi: 10.1146/annurev-biochem-060614-034402, PMID: 25621509 PMC4452390

[6] Mitchell RA Rodney B. & Burgess, A. W. Epidermal growth factor receptor: Structure-function informing the design of anticancer therapeutics. Exp Cell Res. (2018) 371:1–19. doi: 10.1016/j.yexcr.2018.08.009, PMID: 30098332

[7] DeSantis CE Ma J Gaudet MM Newman LA Miller KD Goding Sauer A. Breast cancer statistics, 2019. CA Cancer J Clin. (2019) 69:438–51. doi: 10.3322/caac.21583, PMID: 31577379

[8] Junttila TT Akita RW Parsons K Fields C Lewis Phillips GD Friedman LS. Ligand-independent HER2/HER3/PI3K complex is disrupted by trastuzumab and is effectively inhibited by the PI3K inhibitor GDC-0941. Cancer Cell. (2009) 15:429–40. doi: 10.1016/j.ccr.2009.03.020, PMID: 19411071

[9] Moasser MM Krop IE. The evolving landscape of HER2 targeting in breast cancer. JAMA Oncol. (2015) 1:1154–61. doi: 10.1001/jamaoncol.2015.2286, PMID: 26204261

[10] Perez EA Thompson EA Ballman KV Anderson SK Asmann YW Kalari KR. Genomic analysis reveals that immune function genes are strongly linked to clinical outcome in the North Central Cancer Treatment Group n9831 Adjuvant Trastuzumab Trial. J Clin Oncol. (2015) 33:701–8. doi: 10.1200/JCO.2014.57.6298, PMID: 25605861 PMC4334774

[11] Agus DB Akita RW Fox WD Lewis GD Higgins B Pisacane PI. Targeting ligand-activated ErbB2 signaling inhibits breast and prostate tumor growth. Cancer Cell. (2002) 2:127–37. doi: 10.1016/S1535-6108(02)00097-1, PMID: 12204533

[12] Baselga J Cortes J Kim SB Im SA Hegg R Im YH. Pertuzumab plus trastuzumab plus docetaxel for metastatic breast cancer. N Engl J Med. (2012) 366:109–19. doi: 10.1056/NEJMoa1113216, PMID: 22149875 PMC5705202

[13] von Minckwitz G Procter M de Azambuja E Zardavas D Benyunes M Viale G. Adjuvant pertuzumab and trastuzumab in early HER2-positive breast cancer. N Engl J Med. (2017) 377:122–31. doi: 10.1056/NEJMoa1703643, PMID: 28581356 PMC5538020

[14] Tabernero J Hoff PM Shen L Ohtsu A Shah MA Cheng K. Pertuzumab plus trastuzumab and chemotherapy for HER2-positive metastatic gastric or gastro-oesophageal junction cancer (JACOB): final analysis of a double-blind, randomised, placebo-controlled phase 3 study. Lancet Oncol. (2018) 19:1372–84. doi: 10.1016/S1470-2045(18)30481-9, PMID: 30217672

[15] Sperinde J Jin X Banerjee J Penuel E Saha A Diedrich G. Quantitation of p95HER2 in paraffin sections by using a p95-specific antibody and correlation with outcome in a cohort of trastuzumab-treated breast cancer patients. Clin Cancer Res. (2010) 16:4226–35. doi: 10.1158/1078-0432.Ccr-10-0410, PMID: 20664024

[16] Vernieri C Milano M Brambilla M Mennitto A Maggi C Cona MS. Resistance mechanisms to anti-HER2 therapies in HER2-positive breast cancer: Current knowledge, new research directions and therapeutic perspectives. Crit Rev Oncol Hematol. (2019) 139:53–66. doi: 10.1016/j.critrevonc.2019.05.001, PMID: 31112882

[17] Stoup N Liberelle M Lebegue N Van Seuningen I. Emerging paradigms and recent progress in targeting ErbB in cancers. Trends Pharmacol Sci. (2024) 45: 552–76. doi: 10.1016/j.tips.2024.04.009, PMID: 38797570

[18] Geuijen CAW De Nardis C Maussang D Rovers E Gallenne T Hendriks LJA. Unbiased combinatorial screening identifies a bispecific igG1 that potently inhibits HER3 signaling via HER2-guided ligand blockade. Cancer Cell. (2018) 33:922–936.e910. doi: 10.1016/j.ccell.2018.04.003, PMID: 29763625

[19] Xu JG Chen S He Y Zhu X Wang Y Ye Z. An antibody cocktail targeting two different CD73 epitopes enhances enzyme inhibition and tumor control. Nat Commun. (2024) 15:10872. doi: 10.1038/s41467-024-55207-9, PMID: 39738003 PMC11685497

[20] Ji D Zhang J Shen W Du Y Xu J Yang J. Preliminary safety, efficacy and pharmacokinetics (PK) results of KN026, a HER2 bispecific antibody in patients (pts) with HER2-positive metastatic breast cancer. J Clin Oncol. (2020) 38:1041–1. doi: 10.1200/JCO.2020.38.15_suppl.1041

[21] Meric-Bernstam F Beeram M Hamilton E Oh DY Hanna DL Kang YK. Zanidatamab, a novel bispecific antibody, for the treatment of locally advanced or metastatic HER2-expressing or HER2-amplified cancers: a phase 1, dose-escalation and expansion study. Lancet Oncol. (2022) 23:1558–70. doi: 10.1016/S1470-2045(22)00621-0, PMID: 36400106

[22] Wang X Lee KS Zeng X Sun T Im Y-H Li H. Zanidatamab (zani), a HER2-targeted bispecific antibody, in combination with docetaxel as first-line therapy (1L) for patients (pts) with advanced HER2-positive breast cancer (BC): Updated results from a phase 1b/2 study. J Clin Oncol. (2023) 41:1044–4. doi: 10.1200/JCO.2023.41.16_suppl.1044

[23] Weisser NE Sanches M Escobar-Cabrera E O'Toole J Whalen E Chan PWY. An anti-HER2 biparatopic antibody that induces unique HER2 clustering and complement-dependent cytotoxicity. Nat Commun. (2023) 14:1394. doi: 10.1038/s41467-023-37029-3, PMID: 36914633 PMC10011572

[24] Wei H Cai H Jin Y Wang P Zhang Q Lin Bai YX. Structural basis of a novel heterodimeric Fc for bispecific antibody production. Oncotarget. (2017) 8:51037–49. doi: 10.18632/oncotarget.17558, PMID: 28881627 PMC5584228

[25] Liu X Fan X Gao X Hu W Sun P. Leveraging HER2-targeted biparatopic antibodies in solid tumors. Pharmacol Res. (2025) 214:107687. doi: 10.1016/j.phrs.2025.107687, PMID: 40054541

[26] Bai X. Computational-aided rational mutation design of pertuzumab to overcome active HER2 mutation S310F through antibody-drug conjugates. Proc Natl Acad Sci U.S.A. (2025) 122:e2413686122. doi: 10.1073/pnas.2413686122, PMID: 39793038 PMC11725927

[27] Tahmasebi F Kazemi T Amiri MM Khoshnoodi J Mahmoudian J Bayat AA. *In vitro* assessment of the effects of anti-HER2 monoclonal antibodies on proliferation of HER2-overexpressing breast cancer cells. Immunotherapy. (2014) 6:43–9. doi: 10.2217/imt.13.156, PMID: 24341883

[28] Lu Q Wang L Zhang Y Yu X Wang C Wang H. An anti-ErbB2 fully human antibody circumvents trastuzumab resistance. Oncotarget. (2016) 7:67129. doi: 10.18632/oncotarget.11562, PMID: 27564098 PMC5341862

[29] Meng Y Zheng L Yang Y Wang H Dong J Wang C. A monoclonal antibody targeting ErbB2 domain III inhibits ErbB2 signaling and suppresses the growth of ErbB2-overexpressing breast tumors. Oncogenesis. (2016) 5:e211. doi: 10.1038/oncsis.2016.25, PMID: 26999718 PMC4815051

[30] Yu X Wang L Shen Y Wang C Zhang Y Meng Y. Targeting EGFR/HER2 heterodimerization with a novel anti-HER2 domain II/III antibody. Mol Immunol. (2017) 87:300–7. doi: 10.1016/j.molimm.2017.05.010, PMID: 28531814

[31] Shu M Yan H Xu C Wu Y Chi Z Nian W. A novel anti-HER2 antibody GB235 reverses Trastuzumab resistance in HER2-expressing tumor cells. Vitro vivo Sci Rep. (2020) 10:2986. doi: 10.1038/s41598-020-59818-2, PMID: 32076029 PMC7031383

[32] Liu X Luan L Liu X Jiang D Deng J Xu J. A novel nanobody-based HER2-targeting antibody exhibits potent synergistic antitumor efficacy in trastuzumab-resistant cancer cells. Front Immunol. (2023) 14:1292839. doi: 10.3389/fimmu.2023.1292839, PMID: 37954614 PMC10634241

[33] Mullin M McClory J Haynes W Grace J Robertson N van Heeke G. Applications and challenges in designing VHH-based bispecific antibodies: leveraging machine learning solutions. MAbs. (2024) 16:2341443. doi: 10.1080/19420862.2024.2341443, PMID: 38666503 PMC11057648

[34] Deng J Geng Z Luan L Jiang D Lu J Zhang H. Novel anti-trop2 nanobodies disrupt receptor dimerization and inhibit tumor cell growth. Pharmaceutics. (2024) 16:1255. doi: 10.3390/pharmaceutics16101255, PMID: 39458590 PMC11510716

[35] Zhao D Liu L Liu X Zhang J Yin Y Luan L. A potent synthetic nanobody with broad-spectrum activity neutralizes SARS-CoV-2 virus and the Omicron variant BA.1 through a unique binding mode. J Nanobiotechnol. (2022) 20:41. doi: 10.1186/s12951-022-01619-y, PMID: 36109732 PMC9479348

[36] Ai Z Wang B Song Y Cheng P Liu X Sun P. Prodrug-based bispecific antibodies for cancer therapy: advances and future directions. Front Immunol. (2025) 16:1523693. doi: 10.3389/fimmu.2025.1523693, PMID: 39911391 PMC11794264

[37] Xu J Sun P Zhu W Liu X Ma L. FGF19 in solid tumors: molecular mechanisms, metabolic reprogramming, and emerging therapeutic opportunities. Theranostics. (2026) 16:345–97. doi: 10.7150/thno.121601 PMC12665127 41328353

[38] Hou J Du K Li J Li Z Cao S Zhang S. Research trends in the use of nanobodies for cancer therapy. J Controlled Release. (2025) 381:113454. doi: 10.1016/j.jconrel.2025.01.045, PMID: 39922288

[39] Madsen AV Kristensen P Buell AK Goletz S. Generation of robust bispecific antibodies through fusion of single-domain antibodies on IgG scaffolds: a comprehensive comparison of formats. MAbs. (2023) 15:2189432. doi: 10.1080/19420862.2023.2189432, PMID: 36939220 PMC10038023

[40] Misson Mindrebo L Liu H Ozorowski G Tran Q Woehl J Khalek I. Fully synthetic platform to rapidly generate tetravalent bispecific nanobody-based immunoglobulins. Proc Natl Acad Sci U.S.A. (2023) 120:e2216612120. doi: 10.1073/pnas.2216612120, PMID: 37276407 PMC10268213

[41] Abramson J Adler J Dunger J Evans R Green T Pritzel A. Accurate structure prediction of biomolecular interactions with AlphaFold 3. Nature. (2024) 630:493–500. doi: 10.1038/s41586-024-07487-w, PMID: 38718835 PMC11168924

[42] Liu X Song Y Cheng P Liang B Xing D. Targeting HER2 in solid tumors: Unveiling the structure and novel epitopes. Cancer Treat Rev. (2024) 130:102826. doi: 10.1016/j.ctrv.2024.102826, PMID: 39270365

[43] Kast F Schwill M Stuber JC Pfundstein S Nagy-Davidescu G Rodriguez JMM. Engineering an anti-HER2 biparatopic antibody with a multimodal mechanism of action. Nat Commun. (2021) 12:3790. doi: 10.1038/s41467-021-23948-6, PMID: 34145240 PMC8213836

[44] Verhaar ER Woodham AW Ploegh HL. Nanobodies in cancer. Semin Immunol. (2020) 52:101425. doi: 10.1016/j.smim.2020.101425, PMID: 33272897 PMC8164649

[45] Madsen AV Kristensen P Goletz S. IgG-VHH bispecific fusion antibodies: challenges and opportunities as therapeutic agents. Expert Opin Biol Ther. (2024) 24:1–4. doi: 10.1080/14712598.2024.2336068, PMID: 38544310

[46] Ding X Gu W Zhong Y Hao X Liu J Xia S. A novel HER2-targeting antibody 5G9 identified by large-scale trastuzumab-based screening exhibits potent synergistic antitumor activity. EBioMedicine. (2020) 60:102996. doi: 10.1016/j.ebiom.2020.102996, PMID: 32950002 PMC7501074

[47] Cheng J Liang M Carvalho MF Tigue N Faggioni R Roskos LK. Molecular mechanism of HER2 rapid internalization and redirected trafficking induced by anti-HER2 biparatopic antibody. Antibodies (Basel). (2020) 9:(2020). doi: 10.3390/antib9030049, PMID: 32961882 PMC7551206

[48] Stuber JC Richter CP Bellon JS Schwill M Konig I Schuler B. Apoptosis-inducing anti-HER2 agents operate through oligomerization-induced receptor immobilization. Commun Biol. (2021) 4:762. doi: 10.1038/s42003-021-02253-4, PMID: 34155320 PMC8217238

[49] Chen Y Lu A Hu Z Li J Lu J. ERBB3 targeting: A promising approach to overcoming cancer therapeutic resistance. Cancer Lett. (2024) 599:217146. doi: 10.1016/j.canlet.2024.217146, PMID: 39098760

[50] Swain SM Shastry M Hamilton E. Targeting HER2-positive breast cancer: advances and future directions. Nat Rev Drug Discov. (2022) 22:1–26. doi: 10.1038/s41573-022-00579-0, PMID: 36344672 PMC9640784

[51] Gupta A Michelini F Shao H Yeh C Drago JZ Liu D. EGFR-directed antibodies promote HER2 ADC internalization and efficacy. Cell Rep Med. (2024) 5:101792. doi: 10.1016/j.xcrm.2024.101792, PMID: 39437778 PMC11604483

[52] Yamashita-Kashima Y Iijima S Yorozu K Furugaki K Kurasawa M Ohta M. Pertuzumab in combination with trastuzumab shows significantly enhanced antitumor activity in HER2-positive human gastric cancer xenograft models. Clin Cancer Res. (2011) 17:5060–70. doi: 10.1158/1078-0432.CCR-10-2927, PMID: 21700765

[53] Li B Meng Y Zheng L Zhang X Tong Q Tan W. Bispecific antibody to ErbB2 overcomes trastuzumab resistance through comprehensive blockade of ErbB2 heterodimerization. Cancer Res. (2013) 73:6471–83. doi: 10.1158/0008-5472.Can-13-0657, PMID: 24046294

[54] Niquille DL Fitzgerald KM Gera N. Biparatopic antibodies: therapeutic applications and prospects. MAbs. (2024) 16:2310890. doi: 10.1080/19420862.2024.2310890, PMID: 38439551 PMC10936611

[55] Tural D Akar E Mutlu H Kilickap S. P95 HER2 fragments and breast cancer outcome. Expert Rev Anticancer Ther. (2014) 14:1089–96. doi: 10.1586/14737140.2014.929946, PMID: 24968823

[56] Gaibar M Beltran L Romero-Lorca A Fernandez-Santander A Novillo A. Somatic mutations in HER2 and implications for current treatment paradigms in HER2-positive breast cancer. J Oncol. (2020) 2020:6375956. doi: 10.1155/2020/6375956, PMID: 32256585 PMC7081042

[57] Yoon J Oh DY. HER2-targeted therapies beyond breast cancer - an update. Nat Rev Clin Oncol. (2024). doi: 10.1038/s41571-024-00924-9, PMID: 39039196

[58] Li H Er Saw P Song E. Challenges and strategies for next-generation bispecific antibody-based antitumor therapeutics. Cell Mol Immunol. (2020) 17:451–61. doi: 10.1038/s41423-020-0417-8, PMID: 32313210 PMC7193592

[59] Nie S Wang Z Moscoso-Castro M D'Souza P Lei C Xu J. Biology drives the discovery of bispecific antibodies as innovative therapeutics. Antib Ther. (2020) 3:18–62. doi: 10.1093/abt/tbaa003, PMID: 33928225 PMC7990219

[60] Paul S Konig MF Pardoll DM Bettegowda C Papadopoulos N Wright KM. Cancer therapy with antibodies. Nat Rev Cancer. (2024) 24:399–426. doi: 10.1038/s41568-024-00690-x, PMID: 38740967 PMC11180426

[61] Silacci M Baenziger-Tobler N Lembke W Zha W Batey S Bertschinger J. Linker length matters, fynomer-Fc fusion with an optimized linker displaying picomolar IL-17A inhibition potency. J Biol Chem. (2014) 289:14392–8. doi: 10.1074/jbc.M113.534578, PMID: 24692552 PMC4022905

[62] Liu X Ma L Li J Sun L Yang Y Liu T. Trop2-targeted therapies in solid tumors: advances and future directions. Theranostics. (2024) 14:3674–92. doi: 10.7150/thno.98178, PMID: 38948057 PMC11209721

[63] Diwanji D Trenker R Thaker TM Wang F Agard DA Verba KA. Structures of the HER2-HER3-NRG1beta complex reveal a dynamic dimer interface. Nature. (2021) 600:339–43. doi: 10.1038/s41586-021-04084-z, PMID: 34759323 PMC9298180

[64] Bai X Sun P Wang X Long C Liao S Dang S. Structure and dynamics of the EGFR/HER2 heterodimer. Cell Discov. (2023) 9:18. doi: 10.1038/s41421-023-00523-5, PMID: 36781849 PMC9925823

[65] Brack S Attinger-Toller I Schade B Mourlane F Klupsch K Woods R. A bispecific HER2-targeting FynomAb with superior antitumor activity and novel mode of action. Mol Cancer Ther. (2014) 13:2030–9. doi: 10.1158/1535-7163.Mct-14-0046-t, PMID: 24994770

[66] Ma J Wang J Xu T Ouyang Q Wang X Wang J. Efficacy and safety of KN026 and docetaxel for HER2-positive breast cancer: a phase II clinical trial. Cancer Commun (Lond). (2025) 45:476–85. doi: 10.1002/cac2.12662, PMID: 39825877 PMC11999887

[67] Escriva-de-Romani S Cejalvo JM Alba E Friedmann J Rodriguez-Lescure A Savard MF. Zanidatamab plus palbociclib and fulvestrant in previously treated patients with hormone receptor-positive, HER2-positive metastatic breast cancer: primary results from a two-part, multicentre, single-arm, phase 2a study. Lancet Oncol. (2025) 26:745–58. doi: 10.1016/S1470-2045(25)00140-8, PMID: 40339592

[68] Xu J Zhao J Chen Y Liu B Du Y Cheng Y. 1425P Efficacy and safety of KN026 in combination with chemotherapy in patients (pts) with unresectable or metastatic HER2 positive gastric or gastroesophageal cancers (GC/GEJC) after first-line treatment with a trastuzumab-containing regimen. Ann Oncol. (2024) 35:S889. doi: 10.1016/j.annonc.2024.08.1491

[69] Li JY Perry SR Muniz-Medina V Wang X Wetzel LK Rebelatto MC. A biparatopic HER2-targeting antibody-drug conjugate induces tumor regression in primary models refractory to or ineligible for HER2-targeted therapy. Cancer Cell. (2016) 29:117–29. doi: 10.1016/j.ccell.2015.12.008, PMID: 26766593

[70] Szymanska M Fosdahl AM Nikolaysen F Pedersen MW Grandal MM Stang E. A combination of two antibodies recognizing non-overlapping epitopes of HER2 induces kinase activity-dependent internalization of HER2. J Cell Mol Med. (2016) 20:1999–2011. doi: 10.1111/jcmm.12899, PMID: 27469139 PMC5020627

[71] Littlefield P Liu L Mysore V Shan Y Shaw DE Jura N. Structural analysis of the EGFR/HER3 heterodimer reveals the molecular basis for activating HER3 mutations. Sci Signaling. (2014) 7:ra114–4. doi: 10.1126/scisignal.2005786, PMID: 25468994 PMC4492339

[72] Waks AG Martinez-Saez O Tarantino P Braso-Maristany F Pascual T Cortes J. Dual HER2 inhibition: mechanisms of synergy, patient selection, and resistance. Nat Rev Clin Oncol. (2024) 21:818–32. doi: 10.1038/s41571-024-00939-2, PMID: 39271787

