# Supplementary material for: Biparatopic HER2-targeted nanobody binder synergizes with trastuzumab in resistant tumor cells
Source: Front Immunol. 2025 Oct 27;16:1711448. doi: 10.3389/fimmu.2025.1711448 (PMC12597947; doi:10.3389/fimmu.2025.1711448)
Supplement: Supplementary file 1 [file DataSheet1.zip › Supplementary Table.docx.DOCX]

Supplementary Material

# Supplementary Table 1. P-values for multiple comparisons of antibody binding (MFI) in tumor cell lines.

| **Antibody concentration (nM)** | **NCI-N87** | | **MCF-7** | | **JIMT-1** | |
| --- | --- | --- | --- | --- | --- | --- |
|  | **Significance** | **P Value** | **Significance** | **P Value** | **Significance** | **P Value** |
| 7.4 | ns | 0.1071 | *** | 0.0004 | **** | <0.0001 |
| 22.2 | ns | 0.0786 | ** | 0.0022 | **** | <0.0001 |
| 66.7 | ns | 0.0981 | *** | 0.0003 | **** | <0.0001 |
| 200 | ns | 0.8017 | *** | 0.0007 | **** | <0.0001 |

2way ANOVA was performed to calculate p-values for comparisons of antibody binding, measured by median fluorescence intensity (MFI), in NCI-N87, MCF-7, and JIMT-1 cells following antibody treatment. Statistical significance was defined as follows: p < 0.0332 (*), p < 0.0021 (**), p < 0.0002 (***), and p < 0.0001 (****).

# Supplementary Table 2. P-values for comparisons of receptor internalization in cancer cell lines

| **Comparison ^a^** | **BT474** | | **NCI-N87** | |
| --- | --- | --- | --- | --- |
|  | **Significance** | **P Value** | **Significance** | **P Value** |
| AH+T vs. AH | **** | <0.0001 | ns | 0.9699 |
| AH+T vs. T | **** | <0.0001 | **** | <0.0001 |
| AH+T vs. P | **** | <0.0001 | **** | <0.0001 |
| AH+T vs. T+P | **** | <0.0001 | **** | <0.0001 |
| AH vs. T | **** | <0.0001 | **** | <0.0001 |
| AH vs. P | **** | <0.0001 | **** | <0.0001 |
| AH vs. T+P | **** | <0.0001 | **** | <0.0001 |
| T vs. P | ** | 0.0015 | ns | 0.993 |
| T vs. T+P | ns | 0.086 | ns | 0.2269 |
| P vs. T+P | ns | 0.2098 | ns | 0.098 |

^a^ One-way ANOVA was used to calculate p-values for comparisons of HER2 internalization following antibody treatment in NCI-N87 and BT474 cells. Statistical significance was defined as follows: p < 0.0332 (*), p < 0.0021 (**), p < 0.0002 (***), and p < 0.0001 (****).

# Supplementary Table 3. P-values for comparisons of percent viability at different treatments in cancer cell lines

| **Cell lines with different treatment** | **Statistical significance** | **Comparison ^a^** | | | | | |
| --- | --- | --- | --- | --- | --- | --- | --- |
|  |  | **AH+T vs. AH** | **AH+T vs. T+P** | **AH+T vs. T** | **AH vs. T+P** | **AH vs. T** | **T+P vs. T** |
| NCI-N87 | Significance | * | **** | **** | *** | **** | ** |
|  | P Value | 0.0109 | <0.0001 | <0.0001 | 0.0001 | <0.0001 | 0.0014 |
| NCI-N87+EGF | Significance | **** | **** | **** | ns | **** | **** |
|  | P Value | <0.0001 | <0.0001 | <0.0001 | 0.9941 | <0.0001 | <0.0001 |
| NCI-N87+HRG | Significance | **** | **** | **** | ns | **** | *** |
|  | P Value | <0.0001 | <0.0001 | <0.0001 | 0.1918 | <0.0001 | 0.0002 |
| SKBR3 | Significance | ns | ns | **** | ns | **** | **** |
|  | P Value | 0.1594 | 0.0738 | <0.0001 | 0.9845 | <0.0001 | <0.0001 |
| SKBR3+EGF | Significance | **** | **** | **** | *** | **** | **** |
|  | P Value | <0.0001 | <0.0001 | <0.0001 | 0.0002 | <0.0001 | <0.0001 |
| SKBR3+HRG | Significance | **** | **** | **** | ns | ** | ** |
|  | P Value | <0.0001 | <0.0001 | <0.0001 | >0.9999 | 0.0035 | 0.004 |
| BT474 | Significance | ns | *** | **** | * | **** | ** |
|  | P Value | 0.3772 | 0.001 | <0.0001 | 0.0147 | <0.0001 | 0.007 |
| BT474+EGF | Significance | ns | **** | **** | ** | **** | * |
|  | P Value | 0.1376 | <0.0001 | <0.0001 | 0.0023 | <0.0001 | 0.0272 |
| BT474+HRG | Significance | ns | ns | ** | ns | ** | * |
|  | P Value | 0.7279 | 0.4524 | 0.001 | 0.9856 | 0.0058 | 0.0121 |

^a^ One-way ANOVA was used to calculate p-values for comparisons of percent cell viability following antibody treatment at 150 nM in NCI-N87 and BT474 cells, or 75 nM in SKBR3 cells. Statistical significance was defined as follows: p < 0.0332 (*), p < 0.0021 (**), p < 0.0002 (***), and p < 0.0001 (****).

# Supplementary Table 4. Hydrogen bonding contacts between anti-HER2 nanobodies and HER2-ECD

| **Nanobody** | **Epitope on HER2-ECD** | **Nanobody residue** | **Distance (Å)** |
| --- | --- | --- | --- |
| A9F5 | LYS 228[ NZ ]^a^ | THR 33[ OG1] | 3.10 |
|  | CYS 244[ N ] | TYR 101[ OH ] | 2.93 |
|  | ARG 98[ NH2] | ASP 106[ OD2] | 2.88 |
|  | ARG 143[ NH2] | SER 107[ OG ] | 3.77 |
|  | ARG 100[ NH2] | SER 108[ OG ] | 2.99 |
|  | SER 214[ OG ] | VAL 110[ O ] | 2.78 |
|  | ASP 234[ OD2] | TYR 99[ OH ] | 2.98 |
|  | SER 209[ O ] | ARG 109[ NH1] | 3.71 |
| H2F5 | LYS 228[ NZ ] | TYR 109[ OH ] | 3.15 |
|  | LYS 228[ O ] | TYR 109[ N ] | 2.15 |

^a^ The atoms involved in hydrogen bonding contact were speciﬁed in parentheses.
