# Supplementary figures and images for: Biparatopic HER2-targeted nanobody binder synergizes with trastuzumab in resistant tumor cells
Source: Front Immunol. 2025 Oct 27;16:1711448. doi: 10.3389/fimmu.2025.1711448 (PMC12597947; doi:10.3389/fimmu.2025.1711448)

Figure.1G

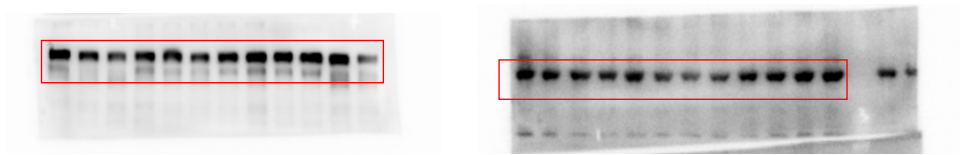

Figure.2A

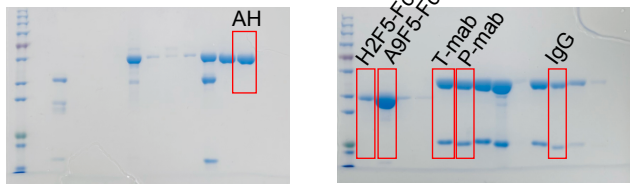

Figure.2B

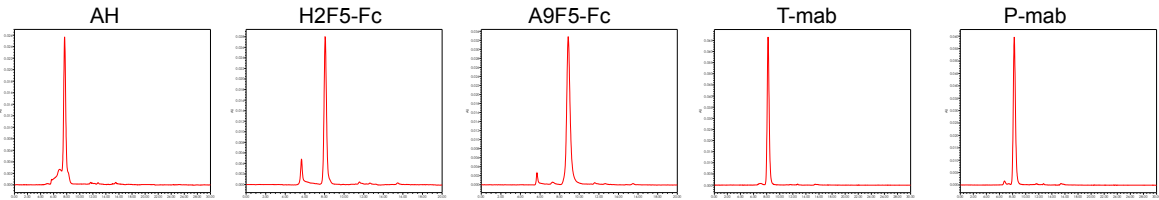

Figure.3C

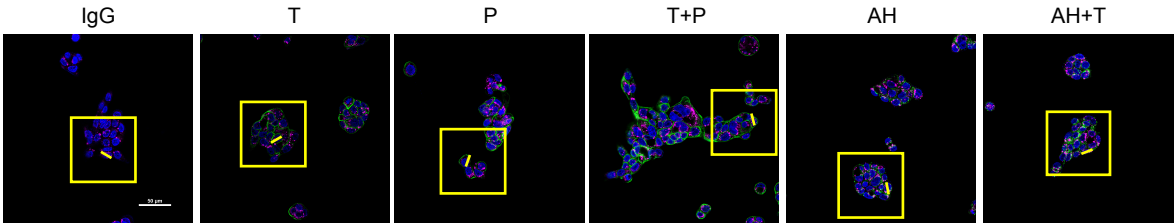

Figure.3D

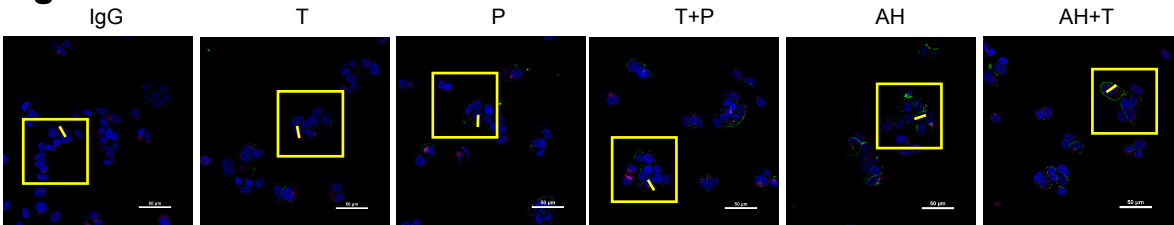

Supplement: Supplementary file 1 [file DataSheet1.zip › Source_data_1.pdf.PDF]
